# A Transcriptome Analysis of mRNAs and Long Non-Coding RNAs in Patients with Parkinson’s Disease

**DOI:** 10.3390/ijms23031535

**Published:** 2022-01-28

**Authors:** Michele Salemi, Giuseppe Lanza, Maria Paola Mogavero, Filomena I. I. Cosentino, Eugenia Borgione, Roberta Iorio, Giovanna Maria Ventola, Giovanna Marchese, Maria Grazia Salluzzo, Maria Ravo, Raffaele Ferri

**Affiliations:** 1 Oasi Research Institute-IRCCS, 94018 Troina, Italy; glanza@oasi.en.it (G.L.); fcosentino@oasi.en.it (F.I.I.C.); eborgione@oasi.en.it (E.B.); msalluzzo@oasi.en.it (M.G.S.); rferri@oasi.en.it (R.F.); 2 Department of Surgery and Medical-Surgical Specialties, University of Catania, 95123 Catania, Italy; 3 Istituti Clinici Scientifici Maugeri, IRCCS, 27100 Pavia, Italy; paola_mogavero@libero.it; 4 Genomix4Life Srl, 84081 Baronissi, Italy; roberta.iorio@genomix4life.com (R.I.); giovanna.ventola@genomix4life.com (G.M.V.); giovanna.marchese@genomix4life.com (G.M.); maria.ravo@genomix4life.com (M.R.); 5 Genome Research Center for Health—CRGS, 84081 Baronissi, Italy

**Keywords:** mRNAs, long non-coding RNAs, RNA sequencing, transcriptome analysis, Parkinson’s disease, inverse comorbidity

## Abstract

Parkinson’s disease (PD) is the second most common neurodegenerative disorder. The number of cases of PD is expected to double by 2030, representing a heavy burden on the healthcare system. Clinical symptoms include the progressive loss of dopaminergic neurons in the substantia nigra of the midbrain, which leads to striatal dopamine deficiency and, subsequently, causes motor dysfunction. Certainly, the study of the transcriptome of the various RNAs plays a crucial role in the study of this neurodegenerative disease. In fact, the aim of this study was to evaluate the transcriptome in a cohort of subjects with PD compared with a control cohort. In particular we focused on mRNAs and long non-coding RNAs (lncRNA), using the Illumina NextSeq 550 DX System. Differential expression analysis revealed 716 transcripts with padj ≤ 0.05; among these, 630 were mRNA (coding protein), lncRNA, and MT_tRNA. Ingenuity pathway analysis (IPA, Qiagen) was used to perform the functional and pathway analysis. The highest statistically significant pathways were: IL-15 signaling, B cell receptor signaling, systemic lupus erythematosus in B cell signaling pathway, communication between innate and adaptive immune cells, and melatonin degradation II. Our findings further reinforce the important roles of mitochondria and lncRNA in PD and, in parallel, further support the concept of inverse comorbidity between PD and some cancers.

## 1. Introduction

Parkinson’s disease (PD) affects ~2–3% of people over 65 years of age and is the second most common neurodegenerative disorder [1,2,3]. Worldwide estimates of PD incidence range from 5 to >35 new cases per 100,000 individuals yearly [4,5,6], and the number of patients is expected to double between 2005 and 2030, thus, representing a heavy burden on society and healthcare system [7,8]. The main pathogenetic feature of PD is the progressive loss of dopaminergic neurons in the substantia nigra of the midbrain, which leads to striatal dopamine deficiency and, subsequently, causes motor dysfunction [7,9]. Main clinical symptoms are resting tremor, rigidity, bradykinesia, and posture instability [10,11], although non-motor symptoms are frequent and disabling, such as cognitive impairment until dementia, autonomic dysfunction, sleep disorders, depression, hyposmia, and behavioral-emotional changes [12,13,14,15]. At present, PD diagnosis mainly relies on clinical manifestations, and no effective disease-modifying treatment strategies exist. Therefore, it is necessary to further explore the pathogenesis of PD, improve the early diagnosis, and design innovative, evidence-based treatments.

A series of recent reports have shown that a series of neurobiological pathways and processes are involved in the molecular pathogenesis of PD, such as oxidative stress, mitochondrial dysfunction, protein degradation, autophagy, axonal transport, calcium homeostasis, and neuroinflammation, which suggest that the onset and course of PD represent a complex systematic and multilevel process [16,17]. Accumulating evidence demonstrates that long non-coding RNAs (lncRNAs) affect the pathogenesis of several diseases, such as cancers, immunological diseases, and neurodegenerative disorders, including Alzheimer’s disease [18] and PD [19,20,21,22,23,24].

Next-generation sequencing provides a high-throughput method for exploring the diverse, polyadenylated RNA populations. This approach allows accurate identification and quantitation of mRNAs and other non-coding RNAs, such as lncRNAs. By definition, lncRNAs are a class of ncRNAs that are longer than 200 nucleotides. RNA polymerase II often transcribes lncRNAs from genomic loci characterized by chromatin states similar to those of mRNA-encoding loci; lncRNAs also share structural features with mRNAs (i.e., 5′-capping, alternative splicing, and 3′-polyadenylation) [25,26,27]. Currently, lncRNAs play a pivotal role in various biological processes, including the regulation of gene expression at both transcriptional and post-transcriptional levels, thus, shaping chromatin conformation and imprinting the genomic loci [28,29,30]. Of note, only a small number of lncRNAs have been functionally characterized, with most of them regulating various aspects of gene expression [31]. Many lncRNAs have been shown to regulate important cancer hallmarks, including apoptosis and proliferation or drug-resistance [32]. In addition, lncRNAs contribute to the complex system organization and gene regulatory networks of the central nervous system, thus, affecting brain patterning, neural stem cell maintenance, stress responses, neurogenesis, glycogenesis, and both neural and synaptic plasticity.

In addition to studying PD in patients, animal and cellular models have also been commonly employed, including neurotoxin-based animal models, transgenic animals over-expressing α-synuclein, and cellular models generated by treatment with methl-4-phenylpyridinium in SH-SY5Y cells [33]. Although animal and cellular models are unable to completely replicate the pathological changes in human PD, the results might provide basic information regarding the mechanisms of PD pathogenesis and the regulatory function of lncRNAs in PD progression, thus, offering new strategies for its diagnosis and treatment. Indeed, high-throughput RNA sequencing and microarray screening results have shown that numerous lncRNAs are differentially expressed in brain tissues and the peripheral blood of patients and animal or cell models of PD, thus, confirming the important role played by lncRNAs [33]. However, overall, in the available studies, the lncRNA expression profiles in PD patients varied considerably and showed poor consistency between cases, probably due to the difference in samples and detection methods. Nevertheless, differently from the invasive brain tissue biopsy, lncRNAs derived from the peripheral blood may be considered as a potentially valuable, diagnostic biomarker for PD, possibly also useful for monitoring its treatment [33].

In this study, a systematic analysis of mRNAs and lncRNAs expression, followed by functional analysis of the results, was performed on the whole gene expression in a cohort of Sicilian patients with PD compared with age-matched, healthy controls. Specifically, the purpose of this study was to evaluate the pathways, genes, and lncRNAs that are particularly dysregulated and that characterize Parkinson’s disease in the cohort we enrolled.

## 2. Results

### 2.1. NGS Transcriptome Analysis of Transcripts

Coding and non-coding, polyadenylated RNA expression profiling was performed by next-generation sequencing in PD patients and CTRL to evaluate their possible deregulation in PD. After filtering out low-quality reads and trimming the adaptors, more than 1200 million sequences were obtained by RNA-seq analysis for the 107 samples sequenced. The reads were aligned against the human genome reference (hg38), paying particular attention to polyadenylated lncRNA identification according to GENCODE gene annotation, the largest manually curated catalog of human lncRNAs.

More than 27,000 RNA molecules, considering both coding and polyadenylated, non-coding ones, were identified in the investigated samples. Among these, 57.55% (15,955) were classified as protein-coding, 20.70% (5739) as lncRNA, and 21.74% (6029) as other molecules. Most of the lncRNAs expressed within the investigated samples belonged to two main categories: long, intergenic, non-coding RNA (lincRNAs, ~40%) and antisense transcripts (~36%).

**Figure 1:**
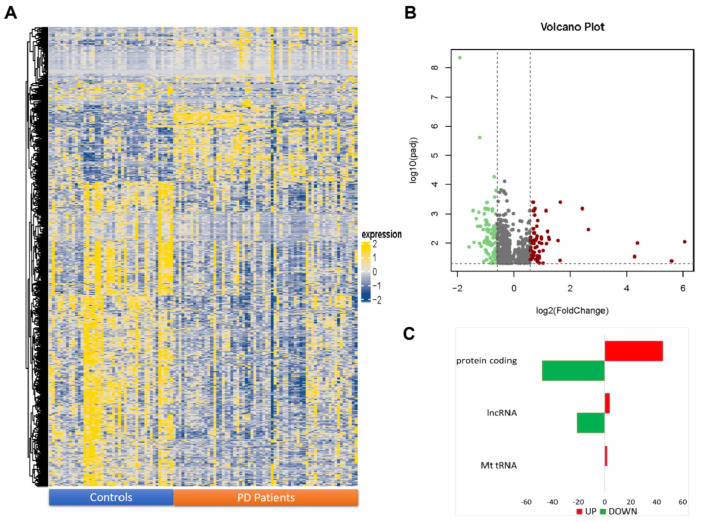
RNA profiling by RNA−seq. (**A**) Heatmap showing the relative expression of 716 RNAs with padj ≤ 0.05 in PD patients compared to controls. The expression value of each RNA was log2-transformed and centered on the median value. Expression values lower or higher than the median are shown in blue or yellow, respectively. (**B**) Volcano plots showing the differentially expressed genes identified (padj ≤ 0.05 and |FC| ≥ 1.5). (**C**) Hystogram showing the gene biotype classification of differentially expressed genes. Red: over-expressed transcripts; green: down-expressed transcripts.

Differential expression analysis revealed 716 transcripts with padj ≤ 0.05 (Figure 1A); among these, 630 were mRNA (coding protein), lncRNA, and MT_tRNA (Supplementary Tables S1 and S2). Specifically, 103 transcripts were also found with FC ≤ −1.5 and 63 with FC ≥ 1.50 in PD subjects compared to controls (Figure 1B). In detail: among the differentially expressed transcript, 48 protein-coding mRNAs and 21 lncRNAs (Figure 1C) were down-expressed (Table 1); and 45 protein-coding mRNAs, 4 lncRNAs, and 2 mitochondrial tRNA (MT_tRNA) (Table 2) were over-expressed in PD subjects compared to controls. The full list of statistically significant, differentially expressed RNAs is available at ArrayExpress (E-MTAB-11326).

### 2.2. Functional and Pathway Analysis of Differentially Expressed Genes

**Figure 2:**
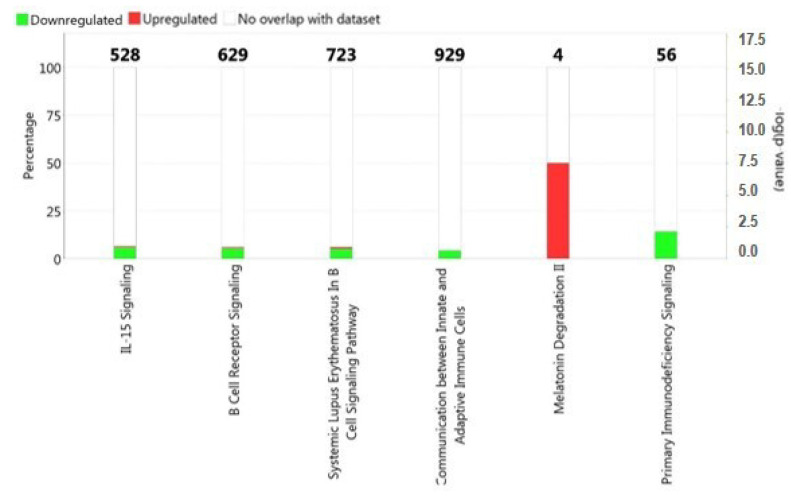
Functional annotation analysis performed on deregulated transcripts (padj ≤ 0.05 and |FC| ≥ 1.5) by ingenuity pathway software (IPA). Red: over−expressed transcripts; green: down−expressed transcripts.

In this study, we focused on the analysis of mRNA (coding protein), lncRNA, and MT_tRNA with padj ≤ 0.05 and |FC| ≥ 1.50. The enrichment analysis of the results revealed some pathways in which differentially expressed RNAs were involved (Figure 2). In particular, the pathways that showed greater significance with a *p*-value < 0.05 were: IL-15 signaling, B cell receptor signaling, systemic lupus erythematosus in B cell signaling pathway, communication between innate and adaptive immune cells, and melatonin degradation II (Figure 2).

**Figure 3:**
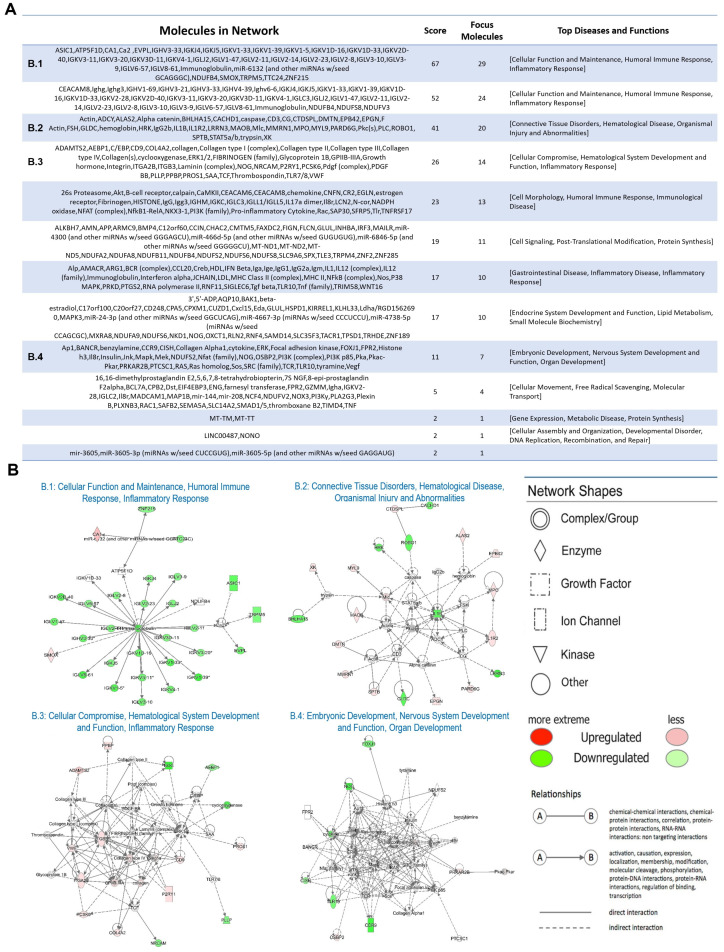
Functional network analysis by ingenuity pathway analysis (IPA). (**A**) The functional network analysis was performed on deregulated transcripts (padj ≤ 0.05 and |FC| ≥ 1.5). In the panel, (**A**) represents all networks identified. (**B**) The IPA network associated with cellular function and maintenance, humoral immune response, inflammatory response (B.1), connective tissue disorders, hematological disease, organismal injury and abnormalities (B.2), cellular compromise, hematological system development and function, inflammatory response (B.3) and embryonic development, nervous system development and function, and organ development (B.4) is shown. Genes are represented by nodes with their shape representing the type of molecule/functional class, and the relationship between the nodes are indicated by edges. Nodes in red are up-regulated in PD, and green color shows down-regulation. The legend explains node shape and edge type.

Moreover, in order to obtain an integrative view of the PD-associated transcripts identified by NGS, functional network analysis was performed by IPA tools, as described in Materials and Methods. This analysis led to the identification of 13 networks, as shown in Figure 3A. Several diseases and biofunctions were found to be enriched, such as cellular function and maintenance, the humoral immune response, inflammatory response, and nervous system development and function (Figure 3B).

## 3. Discussion

Our transcriptome analysis revealed the involvement of interleukin-15 (IL-15), the B cell receptor signal, the communication between innate and adaptive immune cells, and the B cell signaling pathway in systemic lupus erythematosus (SLE).

In recent years, a correlation has emerged between some systemic autoimmune diseases (SLE and antiphospholipid antibody syndrome) and movement disorders such as chorea or parkinsonism, which has led to a growing interest in studying the interconnections between immune-mediated brain disorders and the basal ganglia. Although the pathogenesis is not yet well understood, vasculitic processes at the level of the basal ganglia, mediated by factors still under study [34], seem to be involved. More recently, a large case study of 12,817 patients with SLE found that patients with SLE had a reduced risk of later onset of PD, through a mechanism not well understood, although the authors hypothesized that the immunomodulatory therapy used in SLE patients could be a possible explanation for this inverse comorbidity mechanism [35].

IL-15 is a pro-inflammatory cytokine produced by activated blood monocytes, macrophages, dendritic cells, and activated glial cells (main regulators of both innate and adaptive immune responses in the central nervous system) that promotes T cell proliferation, induction of natural killer and cytotoxic cells, and stimulates B cells to proliferate and secrete immunoglobulins. Finally, IL-15 can be involved in inflammatory reactions and in the activation of the microglia of some common disorders of the central nervous system, including PD [36]. The fact that this cytokine is significantly involved in our series of patients with PD is proof of an important involvement of the immune processes in this disorder, such as activating the microglia and the B cell response.

Emerging evidence suggests that cells involved in the immune system contribute to the pathogenesis of neurodegenerative diseases such as PD; however, it is unclear whether the observed changes in adaptive immunity are causal or secondary to the onset of the disorder [37,38]. HLA DRA and HLA-DRB1 variants are associated with PD [39,40]; also, specific T cells for α-synuclein could be involved in the pathogenesis of the disorder [41], while the role of B cells is not yet clearly understood because they have not yet been found in the brain of patients with PD. There is only evidence on the accumulation of IgG in dopaminergic neurons [42], and the expression of LRRK2 (a gene linked to familial PD) is increased in lymphocytes B in patients with PD [43].

In the evaluation of the transcriptome in our patients with PD, on the other hand, statistical significance was not found for other molecules commonly implicated in the disease, such as dopamine receptors, indicative of a possible primary inflammatory and immune pathogenesis of PD, even before an involvement of the dopaminergic system. Furthermore, no statistical significance was found for other cytokines (IL-6, IL-10, IL-17) implicated in the involvement of T lymphocytes, which could indicate an activation of these cells in PD only later and a preponderant role of lymphocytes B. This might suggest an inflammatory pathogenesis of PD in response to an external stimulus (in genetically predisposed subjects), with a subsequent cascade activation of the immune system and damage to the dopaminergic circuits of the basal ganglia sensitive to this type of cellular response.

An involvement of melatonin in patients with PD has also been reported; this neuro-hormone has a neuroprotective, anti-inflammatory, and antioxidant action and is important for the regulation of circadian functions and the sleep–wake cycle [44,45,46]. In recent years, evidence has emerged on the usefulness of melatonin for the treatment of PD, although the role of this peptide in neurodegenerative diseases is still being studied; these data are very important considering the high prevalence of sleep disorders (such as insomnia, excessive daytime sleepiness, circadian rhythm disturbances, and REM sleep behavior disorder or RBD) in patients with PD [47].

Some studies have shown a positive effect of melatonin in the treatment of insomnia in patients with PD, reporting a significant improvement in sleep quality (as assessed by the Pittsburgh sleep quality index scale) at the end of treatment [48,49]; a more objective evaluation of sleep parameters, through the use of polysomnography, also highlighted an increase in total sleep time and a reduction in the latency of falling asleep after a short period of treatment [49]. Furthermore, melatonin appears to have a beneficial effect in the treatment of excessive daytime sleepiness in patients with PD [49,50,51], although further studies are needed to evaluate the dosage and duration of treatment.

In recent years, a possible role of melatonin has also emerged in the treatment of RBD, a REM sleep parasomnia very often associated with PD, which can frequently represent an onset symptom [52]; melatonin seems to be effective in some cases in the treatment of this disorder, with an excellent tolerance profile, although the trials conducted on the subject are few, with conflicting results [50,53,54].

On the other hand, a connection has recently been shown between melatonin and the dysfunction of mitochondria [55] which, as described above, seem to be involved also in the pathogenesis of PD. Moreover, melatonin and other peptides involved in the regulation of the sleep–wake cycle, such as orexin, play a role in neurodegenerative processes through mechanisms that significantly involve mitochondrial reactions [44,55,56].

There are only a few studies in the literature on the evaluation of the role of melatonin in the pathogenesis of PD [57], although it seems that this neuropeptide is implicated in an important way in the pathology; the finding, in our study, of a significant involvement of melatonin in PD, therefore, adds an important element to the evidence reported by the recent literature.

**Figure 4:**
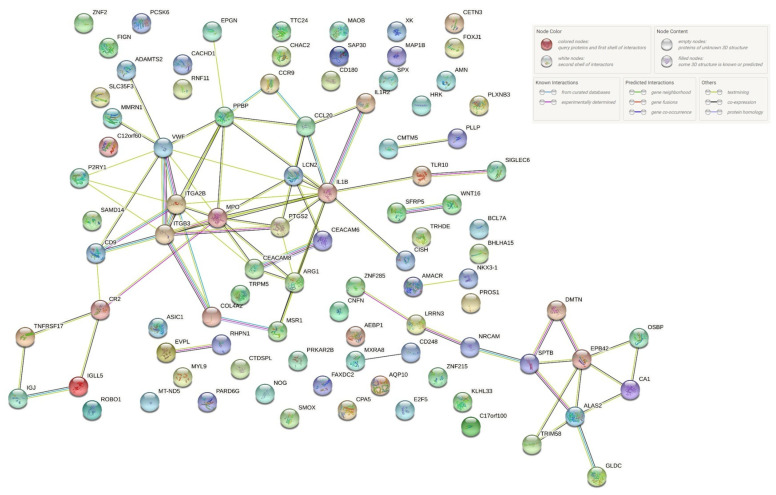
Analysis of interprotein interaction network using the STRING database. Differentially expressed transcripts were analyzed using the STRING interactome, identifying functions and protein–protein interaction networks. The interactions include direct (physical) and indirect (functional) associations. Each edge color indicates a different method of protein–protein interaction prediction, as indicated in the figure legend.

We performed interprotein interaction network analysis using the STRING database. This allowed us to show how differentially expressed genes (Table 1 and Table 2) belonging to the various pathways identified in the study can interact with each other. This highlights how the various pathways are strongly correlated (Figure 4).

The first gene found to be particularly down-regulated in our studies was the NOG gene (Supplementary Table S1). The analysis of NOG gene expression in the rat suggests that it may have a role in both embryonic and adult development. Interestingly, the authors showed increased expression at the brain level [58]. Therefore, to focus on the expression of the NOG gene in subjects with PD could be useful as a potential biomarker.

Among the genes found to be significantly under-expressed in our study, of particular interest seems to be the CCL20 gene (Supplementary Table S1). CCL20 belongs to the chemokine family which are a type of small cytokines. CCL20, on the other hand, is over-expressed in esophageal carcinoma, and it has been shown to have a poor prognosis when its expression increases; in general, a crucial role of CCL20 has been demonstrated in tumor malignancy. Moreover, an increased expression of CCL20 is associated with sets of genes related to metastasis formation [59,60].

Another markedly under-expressed gene in PD found with our transcriptome study was CCR9 (Supplementary Table S1). Lin Lu et al. [61] demonstrated that the over-expression of CCR9 might contribute to distant metastasis and poor overall survival in patients with lung adenocarcinoma. In addition, these findings support the possible use of CCR9 as a novel target for the treatment of lung adenocarcinoma [61].

LINC00487 and FGF14-AS2 are among the lncRNAs that our results identified as down-regulated (Supplementary Table S1) and have already been reported in the literature. In particular, LINC00487 was found to be up-regulated in B cells derived from patients with primary Sjögren’s syndrome which is defined as an autoimmune disease [62]. Furthermore, LINC00487 appears to be particularly up-regulated in squamous cell carcinoma of the lung [63]. In view of the fact that LINC00487 was particularly down-regulated in our results, we believe that this is another example of reverse comorbidity between cancer and PD, as already highlighted in the previous literature [64,65,66,67].

Interestingly, FGF14-AS2 behaves differently from LINC00487 in tumors; in fact, in breast cancer, it appears to be down-regulated, in particular contributing to repressing proliferation, invasion, and migration and inducing apoptosis [68].

Furthermore, the data from this study also support the concept that mitochondrial genes are over-expressed in subjects with PD [69]. In the current study we have highlighted that the MT-ND5, MT-TT, and MT-TW RNAs (Supplementary Table S2) are particularly over-expressed.

Although many reports are present the in literature about mtDNA mutations or decreased activity of the oxidative phosphorylation system (OXPHOS), studies that investigate the expression levels of mtDNA genes are very limited in PD [70,71,72]. In particular, Gezen-Ak et al. [72] reported a higher mRNA level of nine subunits of OXPHOS, including MT-ND5, in the PBMCs of PD patients than those of healthy controls. Biochemical deficits in OXPHOS, in particular for the complex I, are well documented in PD patients [73,74], and mutations in the ND5 gene are also described in association with PD cases [75,76]. On the basis of these observations, we hypothesize that the mRNA over-expression of MT-ND5 can be a compensatory mechanism of mitochondria to address the biochemical deficit of OXPHOS. In addition, for the over-expression of MT-tRNAs observed in our PD cases, we hypothesize a compensatory mechanism through an increase in mitochondrial protein synthesis.

Although the generation of ATP is the most recognized function of mitochondria, these organelles play key roles in many other processes, including lipid biosynthesis, cell signaling, modulating cellular calcium levels, apoptosis, and immune response [77,78].

In particular, mitochondria regulate immune cell function by several mechanisms. First, a major feature of many inflammatory processes is activation of immune cells in response to cytokines, lipopolysaccharide, or other ligands. The effects of many of these signals are mediated by the activation of Toll-like receptors (TLRs) on the cell surface. It is now known that mitochondria reactive oxygen species generation can potentiate macrophage TLR activation [79]. Moreover, mitochondrial antiviral signaling proteins (MAVS) are also present in mitochondria which aggregate in their outer membrane. Double-stranded RNA viruses interact with the cytoplasmic helicase RIG-1, which binds to MAVS and then promotes inflammatory and immune gene expression via transcription factor NF-κB and IRFs [80]. Third, inflammation can also induce cellular production of mitochondrial-derived vesicles, which results in presentation of mitochondrial antigens at the cell surface with activation of major histocompatibility complex-dependent signaling [20,79]. Fourth, differentiation of macrophages into pro-inflammatory (M1) and anti-inflammatory (M2) phenotypes is dependent on alterations in mitochondrial bioenergetic function. In fact, M1 macrophages, activated by IFN-γ, exhibit higher aerobic glycolysis and lower oxidative phosphorylation (OXPHOS) and higher nitric oxide (NO). Conversely, M2 macrophages, activated by IL-4 or IL-13, adopt a metabolic program dominated by β-oxidation [80]. The importance of mitochondria in immunity is now clear, but a better understanding of the molecular components of mitochondria is necessary.

The data of our study allow us to demonstrate a clear involvement of these molecules in our series of patients with PD, further clarifying which cells and cellular mediators of the inflammatory type may be specifically involved in the pathogenesis of PD. Emerging data in the recent literature reveal, in fact, as also described in depth in this discussion, a probable involvement of the inflammatory system, although the specific mechanism is not yet known. Our study, therefore, highlights interesting results, with possible use in clinical practice; however, additional validation with a larger case series (if possible, through further investigations conducted in multicenter studies) and a stratification of the type of patients (for example, age of onset, duration of the illness, course, and ongoing therapy) is required. A recent study validated the parameters for mitochondrial DNA and IL-6 in PD; however, in a larger number of cases than ours and in three third-level European centers (with evaluation of different laboratory parameters) [81]. Our study had different objectives, through the analysis of the transcriptome, but it certainly provides results that could be the basis of interesting future studies aimed at large-scale, numerical validation of what has been observed and, therefore, that could further confirm our findings. An emerging problem in the literature is the growing evidence in the field of proteomics in these pathologies, but studies are still few [82].

## 4. Materials and Methods

### 4.1. Patient Selection

A transcriptome analysis on peripheral blood mononuclear cells (PBMCs) was performed by RNA-seq in 107 subjects, including 64 PD patients (39 males and 25 females, mean age 67.57 ± 11.77 years; disease duration 5.68 ± 4.42 years), diagnosed according to the latest diagnostic criteria for PD PMDI [83], and 43 healthy controls (CTRL, 17 males and 26 females, mean age 64.86 ± 14.75 years). The subjects were recruited at the Oasi Research Institute—IRCCS (Troina, Italy). Clinical-demographic characteristics of PD patients, along with main comorbidities and specific drug(s) taken, are summarized in the Supplementary Material. Overall, 27 patients exhibited an akinetic-rigid phenotype, and 10 were tremor-dominant, whereas the remaining subjects (26) showed mixed features; among them, 5 had prevalent, akinetic-rigid symptoms and 4 a more tremor-dominant phenotype. Of note, 22 patients (13 males) had clinical and video-polysomnography evidence of RBD. Cognitively, 11 subjects exhibited an initial/mild cognitive impairment, whereas an overt dementia (of different severity) was present in 9 patients. Twelve subjects showed a chronic or persistent depressive disorder. Finally, most patients had one or more conventional vascular risk factors and were treated with one or more antiparkinsonian drugs. CTRL were drug-free, did not have any history of neurological or psychiatric disorders, and their neurological exam was entirely normal; details are provided in Supplementary Table S3.

Informed consent for study participation was received from patients and subjects enrolled as controls or, when necessary, from their relatives. The study was carried out in accordance with the Declaration of Helsinki of 1964 and its later amendments, and the Ethics Committee of the Oasi Research Institute—IRCCS, Troina (Italy), approved the protocol on 4 May 2021 (2021/05/04/CE-IRCCS-OASI/43).

### 4.2. RNA Extraction

PBMCs separation was performed using Ficoll-Paque (Ficoll Plaque PLUS–GE Healthcare Life Sciences, Piscataway, NJ, USA), and the RNA was extracted using TRIzol reagent (TRIzol Reagent, Invitrogen Life Technologies, Carlsbad, CA, USA), according to the manufacturer’s instructions [84]. The so-obtained RNA was stored at −80 °C until further processing.

### 4.3. RNA Sequencing and Data Analysis

RNA sequencing and data analysis were performed by Genomix4Life Srl (Baronissi, Italy). The yield and quality of RNA were evaluated by NanoDrop One spectrophotometer (NanoDrop) and by TapeStation 4200 (Agilent Technologies 5301 Stevens Creek Blvd Santa Clara, CA USA), respectively. Indexed libraries were prepared from 1 µg/ea purified RNA with TruSeq Stranded mRNA (Illumina) Library Prep Kit according to the manufacturer’s instructions.

For library quantifications, TapeStation 4200 (Agilent Technologies) was used. Indexed libraries were pooled in equimolar amounts with a final concentration of 2 nM.

Illumina NextSeq 550 DX System was used to sequence the pooled samples in a 2 × 75 paired-end format. The raw sequence files generated (fastq files) underwent quality control analysis using FastQC (https://www.bioinformatics.babraham.ac.uk/projects/fastqc/, accessed on 25 December 2021).

Low-quality reads, short reads (≤25 bp), and adaptor sequences were trimmed with cutadapt [23] (v.2.8). Then, the fastq files were mapped on reference genome using the bioinformatics tool STAR (version 2.7.3a) [85], with the standard parameters for paired reads. The reference track was the assembly Human obtained from GenCode (HG38-Release 37 (GRCh38.p13)).

The quantification of genes expressed for each sequenced sample was computed using feature Count algorithm [86]. Script ad hoc in R was used to normalize the data, using negative binomial generalized linear models, considering all genes expressed in the samples, by Bioconductor DESeq2 package [87]. Transcripts showing fold change ≥ 1.50 or ≤−1.50 (|FC| ≥ 1.50), with adjusted *p*-values ≤ 0.05 (padj), were considered as differentially expressed. ComplexHeatmap [27207943 [88]] and ggplot2 [89] package in R were used to perform HeatMaps and volcano plot, respectively.

### 4.4. Functional and Pathways Analysis of Differentially Expressed Genes

Ingenuity pathway analysis (IPA, Qiagen) was used to perform the functional and pathway analysis. In particular, networks, canonical pathways, and diseases and functional enrichment analysis were selected on all coding genes and on polyadenylated lncRNAs with padj ≤ 0.05 and |FC| ≥ 1.50.

## 5. Conclusions

A larger sample is definitely needed to confirm our results. It also appears to be important to evaluate the comorbidities of these patients and the duration of the disease; however, the extensive analysis carried out on the transcriptome of our patients is a strong point of this study. In fact, it has certainly allowed us to detect statistically significant findings among the factors of innate and adaptive immunity, strengthening the hypothesis of the efficacy of immunotherapy in patients with PD, with a possibly neuroprotective effect recently emphasized in the literature [90,91], even during the earliest stages of the disorder. In addition, our findings further reinforce the important roles of mitochondria and lncRNA in PD and provide an additional contribution to the concept of inverse comorbidity between PD and various cancers. In fact, in recent years, many studies have been conducted on the inverse comorbidity between neurodegenerative diseases and tumors; in cancer patients there seems to be a 20–50% lower risk of contracting PD, and patients with PD seem to have a lower incidence of cancer [92]. These data have led to the identification of several drugs used for the treatment of neurodegenerative disorders and cancer, such as galantamine, selegiline, exemestane, and estradiol, as potential modulators of the comorbidity observed between neurodegeneration and cancer [93].

## Figures and Tables

**Table 1:** Protein-coding mRNAs and lncRNAs down-expressed in PD subjects compared to controls (padj ≤ 0.05 and |FC| ≥ 1.5).

RNA	Fold Change	Type	RNA	Fold Change	Type	RNA	Fold Change	Type
NOG	−3.75	pr_coding	EVPL	−1.89	pr_coding	AC009237.14	−1.60	lncRNA
CCL20	−3.02	pr_coding	ROBO1	−1.89	pr_coding	ZNF2	−1.58	pr_coding
LRRN3	−2.73	pr_coding	ZNF285	−1.87	pr_coding	AL442128.2	−1.58	lncRNA
TNFRSF17	−2.36	pr_coding	FOXJ1	−1.87	pr_coding	KLHL33	−1.58	pr_coding
CCR9	−2.31	pr_coding	AC010331.1	−1.79	lncRNA	LIMD1-AS1	−1.57	lncRNA
PTGS2	−2.22	pr_coding	SFRP5	−1.77	pr_coding	CHAC2	−1.56	pr_coding
MXRA8	−2.11	pr_coding	NKX3-1	−1.77	pr_coding	C12orf60	−1.56	pr_coding
IL1B	−2.08	pr_coding	LINC02848	−1.75	lncRNA	TRPM5	−1.56	pr_coding
GLDC	−2.06	pr_coding	WNT16	−1.71	pr_coding	AL034550.2	−1.56	lncRNA
CD248	−2.05	pr_coding	LEF1-AS1	−1.70	lncRNA	AEBP1	−1.55	pr_coding
HRK	−2.03	pr_coding	LINC02132	−1.70	lncRNA	AC103563.7	−1.55	lncRNA
BHLHA15	−1.99	pr_coding	C17orf100	−1.69	pr_coding	ZNF215	−1.54	pr_coding
AL132996.1	−1.98	lncRNA	CR2	−1.67	pr_coding	CISH	−1.53	pr_coding
MAILR	−1.98	lncRNA	IGLL5	−1.67	pr_coding	IL6R-AS1	−1.52	lncRNA
LINC00487	−1.96	lncRNA	NRCAM	−1.67	pr_coding	BCL7A	−1.52	pr_coding
CPA5	−1.94	pr_coding	TLR10	−1.65	pr_coding	SLC35F3	−1.52	pr_coding
CACHD1	−1.94	pr_coding	AC009123.1	−1.65	lncRNA	MIR4458HG	−1.52	lncRNA
LINC02295	−1.93	lncRNA	PLLP	−1.64	pr_coding	SIGLEC6	−1.52	pr_coding
JCHAIN	−1.93	pr_coding	MIR3142HG	−1.64	lncRNA	ZNF667-AS1	−1.51	lncRNA
FGF14-AS2	−1.91	lncRNA	TTC24	−1.62	pr_coding	AMACR	−1.51	pr_coding
AC097634.1	−1.91	lncRNA	RNF157-AS1	−1.62	lncRNA	CNFN	−1.50	pr_coding
ASIC1	−1.90	pr_coding	AMN	−1.61	pr_coding			

Pr_coding = protein-coding; lncRNA = long non-coding RNA.

**Table 2:** Protein-coding mRNAs, lncRNAs and mitochondrial tRNA over-expressed in PD subjects compared to controls (padj ≤ 0.05 and |FC| ≥ 1.5).

RNA	Fold Change	Type	RNA	Fold Change	Type	RNA	Fold Change	Type
MT-TW	66.69	MT_tRNA	OSBP2	1.87	pr_coding	SPTB	1.64	pr_coding
MT-TT	48.33	MT_tRNA	ARG1	1.87	pr_coding	SMOX	1.63	pr_coding
MT-ND5	20.89	pr_coding	CEACAM6	1.87	pr_coding	P2RY1	1.63	pr_coding
CA1	6.24	pr_coding	LCN2	1.86	pr_coding	MPO	1.62	pr_coding
ADAMTS2	5.36	pr_coding	COL4A2	1.82	pr_coding	VWF	1.61	pr_coding
PPBP	3.11	pr_coding	RNF11	1.79	pr_coding	AC132872.2	1.61	lncRNA
IL1R2	2.95	pr_coding	TRHDE	1.78	pr_coding	CTDSPL	1.60	pr_coding
MAP1B	2.39	pr_coding	SAMD14	1.76	pr_coding	PLXNB3	1.59	pr_coding
CEACAM8	2.36	pr_coding	PROS1	1.76	pr_coding	CD9	1.58	pr_coding
ITGB3	2.24	pr_coding	XK	1.75	pr_coding	SPX	1.58	pr_coding
ITGA2B	2.21	pr_coding	ALAS2	1.70	pr_coding	TRIM58	1.56	pr_coding
MAOB	2.05	pr_coding	LINC02701	1.68	lncRNA	MMRN1	1.54	pr_coding
FIGN	2.05	pr_coding	SAP30	1.67	pr_coding	PARD6G	1.53	pr_coding
EPGN	1.98	pr_coding	DMTN	1.66	pr_coding	FAXDC2	1.53	pr_coding
AQP10	1.97	pr_coding	PCSK6	1.65	pr_coding	AC093849.4	1.52	lncRNA
MYL9	1.96	pr_coding	CMTM5	1.65	pr_coding	CRYZL2P-SEC16B	1.51	lncRNA
EPB42	1.87	pr_coding	PRKAR2B	1.64	pr_coding			

Pr_coding = protein-coding; lncRNA = long non-coding RNA; MT_tRNA = mitochondrial tRNA.

## Data Availability

The data presented in this study are available on request from the corresponding author.

## Supplementary Materials

The following supporting information can be downloaded at: https://www.mdpi.com/article/10.3390/ijms23031535/s1.
