# Supplementary material for: A Transcriptome Analysis of mRNAs and Long Non-Coding RNAs in Patients with Parkinson’s Disease
Source: Int J Mol Sci. 2022 Jan 28;23(3):1535. doi: 10.3390/ijms23031535 (PMC8836138; doi:10.3390/ijms23031535)
Supplement: Supplementary file 1 [file ijms-23-01535-s001.zip › ijms-1551323-supplementary.pdf]

Supplementar Table S1. List of underexpressed (|FC| ≥1.50) RNA found in our study.

Underexpressed RNA				
**Gene**	**Fold Change**	**Chromosome**	**Gene**	**RNA type**
**NOG**	-3.75[f]	chr17	ENSG00000183691.6	protein coding
**CCL20**	-3.02[a]	chr2	ENSG00000115009.13	protein coding
**LRRN3**	-2.73[c]	chr7	ENSG00000173114.13	protein coding
**RNA5SP352**	-2.66[b]	chr11	ENSG00000200278.1	rRNA pseudogene
**IGKV1-39**	-2.52[b]	KQ031384.1	ENSG00000282120.1	IG V gene
**IGKV1-39**	-2.52[b]	chr2	ENSG00000242371.1	IG V gene
**IGLV8-61**	-2.43[b]	chr22	ENSG00000211638.2	IG V gene
**TNFRSF17**	-2.36[c]	chr16	ENSG00000048462.11	protein coding
**IGKV2D-40**	-2.32[a]	chr2	ENSG00000251039.2	IG V gene
**CCR9**	-2.31[f]	chr3	ENSG00000173585.16	protein coding
**IGKV1-5**	-2.28[b]	KQ031384.1	ENSG00000282801.1	IG V gene
**IGKV1-5**	-2.28[b]	chr2	ENSG00000243466.1	IG V gene
**IGKV1-33**	-2.26[b]	chr2	ENSG00000242076.2	IG V gene
**IGKV1-33**	-2.26[b]	KQ031384.1	ENSG00000282811.1	IG V gene
**PTGS2**	-2.22[a]	chr1	ENSG00000073756.13	protein coding
**MXRA8**	-2.11[b]	chr1	ENSG00000162576.17	protein coding
**IL1B**	-2.08[a]	chr2	ENSG00000125538.12	protein coding
**IGLC6**	-2.07[b]	chr22	ENSG00000222037.5	IG C pseudogene
**GLDC**	-2.06[b]	chr9	ENSG00000178445.10	protein coding
**CD248**	-2.05[c]	chr11	ENSG00000174807.4	protein coding
**HRK**	-2.03[c]	chr12	ENSG00000135116.9	protein coding
**IGKV1D-16**	-2[a]	chr2	ENSG00000241244.1	IG V gene
**BHLHA15**	-1.99[b]	chr7	ENSG00000180535.4	protein coding
**IGHV3-33**	-1.98[c]	chr14	ENSG00000211955.2	IG V gene
**IGHV3-33**	-1.98[c]	KI270846.1	ENSG00000282642.1	IG V gene
**AL132996.1**	-1.98[b]	chr6	ENSG00000260271.3	lncRNA
**MAILR**	-1.98[b]	chr8	ENSG00000253320.7	lncRNA
**LINC00487**	-1.96[a]	chr2	ENSG00000205837.7	lncRNA
**CPA5**	-1.94[a]	chr7	ENSG00000158525.16	protein coding
**CACHD1**	-1.94[b]	chr1	ENSG00000158966.16	protein coding
**LINC02295**	-1.93[c]	chr14	ENSG00000258511.1	lncRNA
**IGLV1-47**	-1.93[b]	chr22	ENSG00000211648.2	IG V gene
**JCHAIN**	-1.93[b]	chr4	ENSG00000132465.12	protein coding
**FGF14-AS2**	-1.91[a]	chr13	ENSG00000272143.1	lncRNA
**AC097634.1**	-1.91[b]	chr3	ENSG00000270562.1	lncRNA
**ASIC1**	-1.9[b]	chr12	ENSG00000110881.12	protein coding
**EVPL**	-1.89[b]	chr17	ENSG00000167880.8	protein coding
**ROBO1**	-1.89[a]	chr3	ENSG00000169855.20	protein coding
**IGLV3-10**	-1.89[b]	chr22	ENSG00000211669.3	IG V gene
**ZNF285**	-1.87[c]	chr19	ENSG00000267508.6	protein coding
**FOXJ1**	-1.87[a]	chr17	ENSG00000129654.8	protein coding
**IGKV4-1**	-1.85[b]	chr2	ENSG00000211598.2	IG V gene
**IGKV3-11**	-1.85[b]	chr2	ENSG00000241351.3	IG V gene
**IGKV3-11**	-1.85[b]	KQ031384.1	ENSG00000282823.1	IG V gene
**AC234301.1**	-1.81[b]	KI270846.1	ENSG00000278082.3	IG V gene
**AC010331.1**	-1.79[a]	chr19	ENSG00000277383.1	lncRNA
**SFRP5**	-1.77[a]	chr10	ENSG00000120057.5	protein coding
**NKX3-1**	-1.77[a]	chr8	ENSG00000167034.10	protein coding
**IGLC3**	-1.75[a]	chr22	ENSG00000211679.2	IG C gene
**LINC02848**	-1.75[b]	chr7	ENSG00000226581.2	lncRNA
**WNT16**	-1.71[a]	chr7	ENSG00000002745.13	protein coding
**LEF1-AS1**	-1.7[c]	chr4	ENSG00000232021.7	lncRNA
**LINC02132**	-1.7[a]	chr16	ENSG00000268804.1	lncRNA
**C17orf100**	-1.69[c]	chr17	ENSG00000256806.6	protein coding
**IGKJ5**	-1.69[b]	chr2	ENSG00000211593.2	IG J gene
**IGLV2-23**	-1.68[b]	chr22	ENSG00000211660.3	IG V gene
**IGLV2-14**	-1.68[a]	chr22	ENSG00000211666.2	IG V gene
**CR2**	-1.67[b]	chr1	ENSG00000117322.18	protein coding
**IGKV3D-11**	-1.67[a]	chr2	ENSG00000211632.4	IG V gene
**IGLL5**	-1.67[b]	chr22	ENSG00000254709.8	protein coding
**NRCAM**	-1.67[a]	chr7	ENSG00000091129.21	protein coding
**RNU4-62P**	-1.67[a]	chr3	ENSG00000222057.1	snRNA
**IGHM**	-1.66[b]	KI270846.1	ENSG00000282657.3	IG C gene
**IGHM**	-1.66[b]	chr14	ENSG00000211899.10	IG C gene
**TLR10**	-1.65[b]	chr4	ENSG00000174123.11	protein coding
**AC009123.1**	-1.65[a]	chr16	ENSG00000250685.7	lncRNA
**AL390719.1**	-1.65[a]	chr1	ENSG00000217801.10	transcribed unprocessed
**PLLP**	-1.64[b]	chr16	ENSG00000102934.10	protein coding

**MIR3142HG**	-1.64[b]	chr5	ENSG00000253522.6	lncRNA
**IGLJ2**	-1.63[a]	chr22	ENSG00000211676.2	IG J gene
**TTC24**	-1.62[def]	chr1	ENSG00000187862.13	protein coding
**RNF157-AS1**	-1.62[b]	chr17	ENSG00000267128.2	lncRNA
**AMN**	-1.61[c]	chr14	ENSG00000166126.11	protein coding
**AC009237.14**	-1.6[a]	chr2	ENSG00000272913.1	lncRNA
**IGLV2-8**	-1.6[a]	chr22	ENSG00000278196.3	IG V gene
**IGKV3-20**	-1.58[a]	KQ031384.1	ENSG00000282402.1	IG V gene
**IGKV3-20**	-1.58[a]	chr2	ENSG00000239951.1	IG V gene
**ZNF2**	-1.58[b]	chr2	ENSG00000275111.5	protein coding
**IGLV2-11**	-1.58[a]	chr22	ENSG00000211668.2	IG V gene
**AL442128.2**	-1.58[b]	chr13	ENSG00000277767.1	lncRNA
**IGLV3-9**	-1.58[a]	chr22	ENSG00000211670.2	IG V gene
**KLHL33**	-1.58[a]	chr14	ENSG00000185271.9	protein coding
**IGKV2-28**	-1.57[a]	KQ031384.1	ENSG00000282025.1	IG V gene
**IGKV2-28**	-1.57[a]	chr2	ENSG00000244116.3	IG V gene
**LIMD1-AS1**	-1.57[c]	chr3	ENSG00000230530.2	lncRNA
**CHAC2**	-1.56[a]	chr2	ENSG00000143942.5	protein coding
**C12orf60**	-1.56[a]	chr12	ENSG00000182993.5	protein coding
**TRPM5**	-1.56[a]	chr11	ENSG00000070985.13	protein coding
**AL034550.2**	-1.56[b]	chr20	ENSG00000277301.1	lncRNA
**AEBP1**	-1.55[c]	chr7	ENSG00000106624.11	protein coding
**AC103563.7**	-1.55[b]	chr2	ENSG00000233850.1	lncRNA
**ZNF215**	-1.54[a]	chr11	ENSG00000149054.16	protein coding
**IGKC**	-1.54[a]	chr2	ENSG00000211592.8	IG C gene
**CISH**	-1.53[b]	chr3	ENSG00000114737.16	protein coding
**IL6R-AS1**	-1.52[a]	chr1	ENSG00000228013.1	lncRNA
**BCL7A**	-1.52[b]	chr12	ENSG00000110987.9	protein coding
**SLC35F3**	-1.52[b]	chr1	ENSG00000183780.13	protein coding
**MIR4458HG**	-1.52[b]	chr5	ENSG00000247516.8	lncRNA
**SIGLEC6**	-1.52[a]	chr19	ENSG00000105492.16	protein coding
**IGKJ4**	-1.51[a]	chr2	ENSG00000211594.2	IG J gene
**ZNF667-AS1**	-1.51[a]	chr19	ENSG00000166770.11	lncRNA
**IGLV2-5**	-1.51[a]	chr22	ENSG00000253234.1	IG V pseudogene
**AMACR**	-1.51[b]	chr5	ENSG00000242110.8	protein coding
**IGLV6-57**	-1.5[a]	chr22	ENSG00000211640.4	IG V gene
**CNFN**	-1.5[a]	chr19	ENSG00000105427.10	protein coding
**E2F5**	-1.5[b]	chr8	ENSG00000133740.11	protein coding
**RHPN1**	-1.5[b]	chr8	ENSG00000158106.14	protein coding
**CD180**	-1.5[b]	chr5	ENSG00000134061.5	protein coding
**PDE4DIPP6**	-1.5[a]	chr1	ENSG00000231551.8	transcribed unprocessed
**CETN3**	-1.5[b]	chr5	ENSG00000153140.9	protein coding
**CCR12P**	-1.5[b]	chr13	ENSG00000238241.2	transcribed unprocessed
**IGKJ3**	-1.49[a]	chr2	ENSG00000211595.2	IG J gene
**IGLV3-1**	-1.48[a]	chr22	ENSG00000211673.2	IG V gene
**IGLC2**	-1.47[a]	chr22	ENSG00000211677.2	IG C gene
**PPIAP45**	-1.43[a]	chr12	ENSG00000258116.1	processed pseudogene
**TRAV13-2**	-1.41[b]	chr14	ENSG00000211791.2	TR V gene
**IGLV3-19**	-1.4[a]	chr22	ENSG00000211663.2	IG V gene
**MIR3671**	-1.39[a]	chr1	ENSG00000265996.1	miRNA
**AC104581.4**	-1.38[a]	chr17	ENSG00000280046.1	TEC
**ANKRD36BP2**	-1.36[a]	chr2	ENSG00000230006.7	transcribed unprocessed
**TRAV4**	-1.32[a]	chr14	ENSG00000211778.2	TR V gene
**TRAV12-3**	-1.3[a]	chr14	ENSG00000211794.3	TR V gene
**AL031009.1**	-1.29[a]	chr16	ENSG00000278987.1	TEC
**HSD17B7P2**	-1.26[a]	chr10	ENSG00000099251.14	transcribed unprocessed
**SUZ12P1**	-1.14[a]	chr17	ENSG00000264538.6	transcribed unprocessed
**IGKV2-30**	-1.05[a]	KQ031384.1	ENSG00000281933.1	IG V gene
**IGKV2-30**	-1.05[a]	chr2	ENSG00000243238.1	IG V gene
**RNU4-2**	-1.03[b]	chr12	ENSG00000202538.1	snRNA
**RNU5A-1**	-1.03[a]	chr15	ENSG00000199568.1	snRNA
**RNU4-1**	-1.02[b]	chr12	ENSG00000200795.1	snRNA
**PCBP2P2**	1.15[a]	chr8	ENSG00000253341.1	processed pseudogene
**EIF4A1P10**	1.23[a]	chrX	ENSG00000229132.2	processed pseudogene
**EIF4BP6**	1.23[b]	chr7	ENSG00000197258.5	processed pseudogene
**DGKZP1**	1.25[a]	chr13	ENSG00000179611.3	processed pseudogene
**TPI1P1**	1.28[a]	chr1	ENSG00000226415.1	processed pseudogene
**EIF4BP3**	1.29[b]	chr9	ENSG00000224546.2	processed pseudogene
**MIR7848**	1.3[a]	chr8	ENSG00000276961.1	miRNA
**EEF1A1P5**	1.31[a]	chr9	ENSG00000196205.8	processed pseudogene
**GAPDHP1**	1.31[a]	chrX	ENSG00000228232.1	processed pseudogene
**ANXA2P2**	1.33[b]	chr9	ENSG00000231991.4	processed pseudogene

**PTMAP2**	1.35[a]	chr5	ENSG00000197744.5	processed pseudogene
**AC100827.2**	1.35[b]	chr15	ENSG00000260144.1	unprocessed pseudogene
**AC016739.1**	1.35[a]	chr2	ENSG00000218175.2	processed pseudogene
**EEF1DP7**	1.36[a]	chr17	ENSG00000263883.1	transcribed processed
**MIR5094**	1.41[a]	chr15	ENSG00000264966.1	miRNA
**RN7SKP296**	1.44[a]	chr3	ENSG00000223117.1	misc RNA
**MSNP1**	1.45[b]	chr5	ENSG00000251593.1	processed pseudogene
**YWHAZP2**	1.45[a]	chr2	ENSG00000213236.3	processed pseudogene
**EEF1A1P10**	1.46[a]	chr7	ENSG00000243746.1	processed pseudogene
**EEF1A1P6**	1.47[b]	chr7	ENSG00000233476.3	processed pseudogene
**FTLP3**	1.47[b]	chr20	ENSG00000226608.3	processed pseudogene
**XRCC6P2**	1.49[a]	chrX	ENSG00000234825.3	processed pseudogene

Padj: a<0.05; b<0.01; c<0.001; d<0.0001; e<0.00001; f<0.000001; FC: fold change; Gene ID

Supplementar Table S2. List of overexpressed (|FC| ≥1.50) RNA found in our study.

**Overexpressed RNA**				
**Gene**	**Fold Change**	**Chromosome**	**Gene**	**RNA Type**
**MT-TW**	66.69[b]	chrM	ENSG00000210117.1	Mt tRNA
**MT-TT**	48.33[a]	chrM	ENSG00000210195.2	Mt tRNA
**MT-ND5**	20.89[a]	chrM	ENSG00000198786.2	protein coding
**MTCO1P12**	19.42[a]	chr1	ENSG00000237973.1	unprocessed pseudogene
**CA1**	6.24[b]	chr8	ENSG00000133742.14	protein coding
**ADAMTS2**	5.36[c]	chr5	ENSG00000087116.16	protein coding
**RPLP0P2**	3.13[c]	chr11	ENSG00000243742.5	transcribed proc pseudogene
**PPBP**	3.11[a]	chr4	ENSG00000163736.4	protein coding
**IL1R2**	2.95[b]	chr2	ENSG00000115590.14	protein coding
**MAP1B**	2.39[b]	chr5	ENSG00000131711.15	protein coding
**CEACAM8**	2.36[b]	chr19	ENSG00000124469.12	protein coding
**AL355472.1**	2.3[b]	chr1	ENSG00000235605.1	proc pseudogene
**ITGB3**	2.24[a]	chr17	ENSG00000259207.9	protein coding
**ITGA2B**	2.21[c]	chr17	ENSG00000005961.19	protein coding
**MAOB**	2.05[a]	chrX	ENSG00000069535.14	protein coding
**FIGN**	2.05[a]	chr2	ENSG00000182263.14	protein coding
**EPGN**	1.98[a]	chr4	ENSG00000182585.10	protein coding
**AQP10**	1.97[b]	chr1	ENSG00000143595.13	protein coding
**MYL9**	1.96[b]	chr20	ENSG00000101335.10	protein coding
**EPB42**	1.87[a]	chr15	ENSG00000166947.15	protein coding
**OSBP2**	1.87[b]	chr22	ENSG00000184792.16	protein coding
**ARG1**	1.87[a]	chr6	ENSG00000118520.15	protein coding
**CEACAM6**	1.87[a]	chr19	ENSG00000086548.9	protein coding
**LCN2**	1.86[a]	chr9	ENSG00000148346.12	protein coding
**COL4A2**	1.82[a]	chr13	ENSG00000134871.19	protein coding
**RNF11**	1.79[a]	chr1	ENSG00000123091.5	protein coding
**AC025048.6**	1.79[b]	chr17	ENSG00000280852.2	transcribed proc pseudogene
**TRHDE**	1.78[a]	chr12	ENSG00000072657.9	protein coding
**AC106865.1**	1.77[a]	chr4	ENSG00000250771.2	transcribed unprocessed
**SAMD14**	1.76[b]	chr17	ENSG00000167100.15	protein coding
**PROS1**	1.76[a]	chr3	ENSG00000184500.16	protein coding
**GAPDHP65**	1.75[b]	chrX	ENSG00000235587.2	proc pseudogene
**XK**	1.75[b]	chrX	ENSG00000047597.7	protein coding
**TUBBP1**	1.71[b]	chr8	ENSG00000127589.4	transcribed proc pseudogene
**ALAS2**	1.7[a]	chrX	ENSG00000158578.21	protein coding
**LINC02701**	1.68[a]	chr11	ENSG00000250508.1	lncRNA
**SAP30**	1.67[c]	chr4	ENSG00000164105.4	protein coding
**DMTN**	1.66[b]	chr8	ENSG00000158856.18	protein coding
**PCSK6**	1.65[b]	chr15	ENSG00000140479.18	protein coding
**CMTM5**	1.65[a]	chr14	ENSG00000166091.21	protein coding
**PRKAR2B**	1.64[b]	chr7	ENSG00000005249.13	protein coding
**SPTB**	1.64[b]	chr14	ENSG00000070182.21	protein coding
**SMOX**	1.63[a]	chr20	ENSG00000088826.18	protein coding
**P2RY1**	1.63[c]	chr3	ENSG00000169860.7	protein coding
**YWHAZP10**	1.62[b]	chrX	ENSG00000217624.2	proc pseudogene
**MPO**	1.62[a]	chr17	ENSG00000005381.8	protein coding
**RPL24P4**	1.62[a]	chr6	ENSG00000181524.6	proc pseudogene
**VWF**	1.61[b]	chr12	ENSG00000110799.14	protein coding
**AC132872.2**	1.61[c]	chr17	ENSG00000264548.1	lncRNA
**MIR3605**	1.61[a]	chr1	ENSG00000284154.1	miRNA
**CTDSPL**	1.6[b]	chr3	ENSG00000144677.15	protein coding
**PLXNB3**	1.59[a]	chrX	ENSG00000198753.12	protein coding
**CD9**	1.58[a]	chr12	ENSG00000010278.15	protein coding
**SPX**	1.58[a]	chr12	ENSG00000134548.11	protein coding
**TRIM58**	1.56[a]	chr1	ENSG00000162722.9	protein coding
**MMRN1**	1.54[a]	chr4	ENSG00000138722.10	protein coding
**PARD6G**	1.53[a]	chr18	ENSG00000178184.16	protein coding
**FAXDC2**	1.53[a]	chr5	ENSG00000170271.11	protein coding
**GOLGA6L5P**	1.52[b]	chr15	ENSG00000230373.8	transcribed unprocessed
**AC093849.4**	1.52[b]	chr4	ENSG00000288025.1	lncRNA
**CRYZL2P-**	1.51[a]	chr1	ENSG00000254154.8	lncRNA
**AC134407.2**	1.5[a]	chr17	ENSG00000279573.1	TEC
**MSR1**	1.5[b]	chr8	ENSG00000038945.15	protein coding

Padj: a<0.05; b<0.01; c<0.001; d<0.0001; e<0.00001; f<0.000001; FC: fold change; Gene ID
